# Singular Pre-Natal Affliction

**Published:** 1887-03

**Authors:** 


### SINGULAR PRE-NATAL AFFLICTION.

The infant son of a well known citizen of Westfield, N.J., though but just large enough to walk and talk, appears and acts like an intoxicated person. The parents were very exemplary young people, but some months after their marriage the young husband lapsed a little from the path of strict temperance.

One winter evening he went from his home ostensibly " to watch with a sick member of the village lodge." The trusting wife discovered at nine o'clock that her husband had forgotten to purchase meat for breakfast, and she went to the market. As she passed a hotel the sound of a man's voice in song came to her ears. She listened but a moment. There was no mistaking her husband's voice, and, scarcely knowing what she did, she looked in at the bar-room window and saw her husband there in a state of beastly intoxication. The effect upon her may well be imagined.

Some time after this a son was born to the parents—a fine, healthy infant, bright and comely. Several months later, when the child began to walk and talk, they took him to the family physician. The little one could not walk without staggering in a most unseemly and ludicrous manner, and could not lisp baby words without a strong hiccough and hesitation. The doctor, averring that if he had seen such symptoms in an adult he should have pronounced them due to intoxication, and nothing else, with little difficulty obtained an account of the unfortunate maternal impression that provoked the peculiar malady with which the child is afflicted. No line of medical treatment could be of use in such a case, and reluctantly the physician gave up the infant boy to endure his strangely miserable life.

" There is nothing like catalepsy about the case," the doctor explained. " There is no healthier child in town. As near as I can explain it, the child has muscles and nerves in that condition of action which its father showed when the mother's impression of his intoxication was received. There are no fits or convulsions, though a tremor is always present. In spite of this fact there is no mental weakness. There is no co-ordination in the movements of the lower limbs and the hands are almost as bad off. His gait is heavy and insecure, a regular drunken reel or stagger. As to his speech, it is not only incoherent and rambling, but he has all of the phenomena of exhilaration or excitement characteristic of the earlier stages of intoxication. His ideas seem to flow rapidly, and all of the senses are wonderfully acute, but there are the muscular tremblings and the actual shambling gait of the drunkard.

"It is a hopeless case; impossible to cure. That boy, if he lives, will have the continued appearance of drunkenness and it cannot be helped. He is drunk, naturally drunk, and though he may become a great scholar, he will never outgrow this malady."

It is a very rare case, and among its features is the odd fact that alcohol in any form and in any quantity acts on the child like a poison.

